# Parallel shifts in ecology and natural selection in an island lizard

**DOI:** 10.1186/1471-2148-9-3

**Published:** 2009-01-06

**Authors:** Ryan Calsbeek, Wolfgang Buermann, Thomas B Smith

**Affiliations:** 1 Department of Biological Sciences, Dartmouth College, Hanover New Hampshire, 03755, USA; 2 Center for Tropical Research, Institute of the Environment, University of California, Los Angeles, CA 90095, USA; 3 Department of Ecology and Evolutionary Biology, University of California, Los Angeles, CA 90095, USA

## Abstract

**Background:**

Natural selection is a potent evolutionary force that shapes phenotypic variation to match ecological conditions. However, we know little about the year-to-year consistency of selection, or how inter-annual variation in ecology shapes adaptive landscapes and ultimately adaptive radiations. Here we combine remote sensing data, field experiments, and a four-year study of natural selection to show that changes in vegetation structure associated with a severe drought altered both habitat use and natural selection in the brown anole, *Anolis sagrei*.

**Results:**

In natural populations, lizards increased their use of vegetation in wet years and this was correlated with selection on limb length but not body size. By contrast, a die-back of vegetation caused by drought was followed by reduced arboreality, selection on body size, and relaxed selection on limb length. With the return of the rains and recovery of vegetation, selection reverted back to pre-drought pattern of selection acting on limb length but not body size. To test for the impact of vegetation loss on natural selection during the drought, we experimentally removed vegetation on a separate study island in a naturally wet year. The experiment revealed similar inter-annual changes in selection on body size but not limb length.

**Conclusion:**

Our results illustrate the dynamic nature of ecology driving natural selection on *Anolis *morphology and emphasize the importance of inter-annual environmental variation in shaping adaptive variation. In addition, results illustrate the utility of using remote sensing data to examine ecology's role in driving natural selection.

## Background

A central goal in evolutionary biology is to understand the origins of adaptive variation that are linked to ecology. However, the ecological forces that drive natural selection and ultimately shape adaptation are dynamic, and may change across years [1-3]. Consequently, there is growing appreciation that an in depth understanding of adaptation in natural populations requires long-term studies of selection [1,3].

A long-standing assertion is that variation in limb length and body size of *Anolis *lizards in the Greater Antilles arose via natural selection in response to differences in ecology [4,5]. Long limbs increase sprinting speed on broad perching surfaces such as tree-trunks, whereas shorter limbs enhance agility on narrow twigs and branches [6,7]. Previous studies [6,8] have suggested that traits such as running performance in different habitats may be important for predator avoidance and foraging efficiency, and body size variation may be a decisive factor in the strength of competitive interactions [9]. Decades of study have repeatedly demonstrated the functional relationships between morphology and habitat use, particularly how suites of traits are favoured under varying ecological conditions [6,8,10]. Though phylogenetic studies of anoles [11] propose that these ecological mechanisms of diversification are conserved through time, we still lack a test of this hypothesis.

The hypothesis depends on consistency of selection pressures such that diversification occurring at the population level would ultimately translate into species level diversity [12,13]. Long-term work on Darwin's finches [1], another adaptive radiation, suggests that environmental fluctuations may make consistency of selection pressures unlikely. However, experimental examinations of environmental variation and its effect on selection have yet to be performed. Indeed, experimental studies of natural selection on combinations of morphological traits over multiple years remain very rare. Fluctuating selection pressures through time make point estimates of selection difficult to interpret, and consequently, a long-term perspective on selection becomes that much more important. Here we provide data from a four-year study to test the hypothesis that changing environmental conditions alter the nature of natural selection on *Anolis *lizards in the wild. We measured selection on natural and experimental populations, and used remote sensing and field-based measures of precipitation and vegetation changes to link our selection data to ecological change.

## Methods

We studied viability selection (i.e., survival to the end of the breeding season) acting on wild populations of male *A. sagrei *(N = 133, 98, 111, and 148 lizards during 2003–2006 respectively) on Kidd cay, a small island (~1600 m^2^) near Georgetown Exuma, Bahamas, and on an offshore cay (Nightmare cay; ~500 m^2^, N = 98 and 92 during 2005 and 2006 respectively). We studied the natural resident population on Kidd cay during 2003, 2005 and 2006, but during 2004, as part of a different study [14], we experimentally replaced all resident male *A. sagrei *with males from an immediately adjacent site on Great Exuma. Nearly all lizards (ca. 85%) in our study population mature and die in a single year [7,9], so each year we studied selection on a different cohort of lizards (the few surviving individuals were excluded from subsequent year's analyses). Though the agent of selection acting on these lizards is difficult to document unambiguously, previous studies suggest that the bulk of mortality arises through predation [15] and competition over territories [9], both of which may depend on variation in locomotor performance in vegetated habitat [7,16].

### Climate and habitat data

During 2004, we measured lizard perching locations by exhaustively sampling the diameters of vegetation in 59 randomly sampled quadrats (1 m^2^) across the entire study island. Though these measures provided a general description of the vegetation that was available to lizards on the island, they failed to adequately describe the habitat actually used by study animals. In subsequent years, we recorded characteristics of the habitat used by lizards, based on the location (vegetative habitat versus not) of perches used by lizards at first capture [17,18]. *Anolis sagrei *are sedentary and very consistent in their use of habitat, and field observations indicate infrequent movement between alternative habitat types [19]. Thus, these measures are likely to better represent the actual range of habitat use exhibited by individuals during our study. Perching data were recorded for all individuals and for each year (N = 969 individual habitat measurements, including measures from four individuals not used in selection analyses owing to missing morphological data) and are reported here as estimates of inter-annual variation in habitat availability and use by lizards. We supplemented these data by measuring changes in the normalized difference vegetation index (NDVI; a measure of vegetation "greenness").

Rainfall data were collected both on the ground (using a rain gauge; monthly totals) and from remote sensing data. We obtained satellite precipitation-data from the microwave sensors onboard the Tropical Rainfall Mapping Mission (TRMM) [20]. The TRMM products were obtained from the global rainfall algorithm (3B43), combining estimates from the sensors with globally gridded rain-gauge data from NOAA's Climate Prediction Center and the Global Precipitation Climatology Center (GPCC). The TRMM products provide estimates of rainfall at 25 km spatial resolution and monthly temporal resolution, covering the years 1998 to 2006. To compute a time series, we extracted rainfall data from the pixel with center coordinate closest to our study region, and aggregated the monthly values to seasonal totals of rainfall estimates.

To compute a time series of vegetation activity for our study region, we obtained normalized difference vegetation index (NDVI) data from the satellite MODIS sensor which were produced by the Global Landcover Facility [21]. The NDVI data set has a spatial resolution of 250 m and a temporal resolution of 16 d and spans the years 2001 to 2006. The NDVI is computed as the difference between near-infrared and red reflectance of the land surface, normalized by the sum of the reflectances, and is indicative of photosynthetic activity.

Considerable residual cloud effects were present in the NDVI data. To improve quality, we first cut the NDVI data for a 5 km^2 ^area with center coordinate W75.555 and N23.500 close to the vicinity of our study region. We then aggregated the 250 m resolution data to 500 m by taking the maximum NDVI within the 2 × 2 subpixel array for each 16 d period. Thereafter, we computed monthly NDVI composites by taking the maximum NDVI within two consecutive 16 d periods. In the subsequent spatial averaging within the study region for each month, we only included 500 m pixels if their NDVI values were above a threshold (NDVI > 0.5) for at least 9 months in a given year. Since NDVI stays high over land throughout the year due to predominant evergreen forest type, the last step ensured that spatial averaging involved almost exclusively land areas within our study region, and excluded small off-shore islands that could not be resolved entirely with the resolution of the MODIS data. In the resulting monthly NDVI time series, spurious effects of cloud cover were still present, and we computed seasonal means by extracting the maximum NDVI from the 3 consecutive months.

### Natural selection, non-manipulated island

Initial lizard captures took place each spring from late May to early June. Each year we attempted to capture all male lizards on our natural study site. Lizards were sexed (males have enlarged post-anal scales), weighed (nearest 0.1 g) and measured snout-vent-length (SVL; nearest 0.5 mm) using a small metal ruler. We recorded habitat use, including whether lizards were first sighted on the ground or in vegetated habitat, as well as the diameter of the perch [9]. Hind and forelimb lengths were measured with dial callipers from the point of insertion into the abdomen to the femoral-tibial and humero-radio-ulnar joints. All measurements were made in the afternoon by a single observer (RC). Lizards were injected with unique combinations of coloured elastomer dye in the ventral side of the hind and forelimbs [22]. Tags serve as permanent identification in the wild, allowing us to track the fate of every individual over the course of the study. Lizards captured at our natural site were released within four hours to their original point of capture. Each year, we attempted to track the fate of every individual over the four-month period that spans the breeding season (June-September).

### Experimental reduction of vegetation

To simulate the effects of drought on vegetation structure during a naturally wet year (2005), we removed approximately 30 percent of the under-story vegetation and 10 percent of the canopy cover from a small off-shore cay adjacent to our natural study island. We used hedge-clippers and machetes to trim green vegetation from the island, and this vegetation was taken away by boat. NDVI has a functional relationship to the fraction of absorbed photosynthetically active radiation (FPAR) and can sometimes be used to calculate changes in net primary productivity [23]. However, the mixture of overstory and understory vegetation at our study site made it difficult to quantify the gains and losses of green biomass due to drought from remotely-sensed data, and we therefore estimated these values qualitatively for our experimental islands. We then introduced a population of 98 individually-marked juvenile male lizards to the experimental island from a site immediately adjacent to our natural study site (for details, see [9]), and measured viability selection over the same four-month period as on Kidd cay. During 2005 and 2006, lizards used in our experimental vegetation manipulation were captured from a site on Great Exuma, 1000 m from our natural study site, and were introduced to Nightmare cay, a small offshore cay currently being used as part of a long-term study of natural selection [9]. The experimental population was introduced at a natural density (~0.3 lizards/m^2^) [24], and the offshore cay was similar in most aspects to our natural site. The lizards on the two islands are also exposed to similar avian predators, (particularly mockingbirds, *Mimus polyglottos*, and herons, *Butorides striatus*) though our natural site also supports a terrestrial lizard that may occasional eat *A. sagrei *(the whiptail lizard *Ameiva*) and which is not present on the offshore cay. We had previously cleared this cay of all naturally occurring lizards to prevent introduced lizards in our study from interacting with native residents. Naturally occurring females on the off-shore cay were not manipulated.

After four months, we estimated the strength and form of natural selection by exhaustively recapturing nearly all surviving lizards on the islands (recapture rates of 97–99% of survivors; we failed to catch at least two male lizards during 2005 and at least one male evaded recapture during the other years). Fall censuses can be considered reliable estimates of survival since emigration from islands is likely to be rare, except during major storms [25], none of which occurred during this study. Census efficiency on islands was determined by regressing the number of lizards captured each day (log transformed) against the cumulative days of capture effort. We then estimated the number of lizards that would have been caught with one more day of the census. This number was less than three lizards per island in all years of study.

### Selection analyses

We used general linear models to extract selection gradients [26,27] for linear (β) and non-linear (γ_1,1 _and γ_1,2_) forms of selection. Linear and non-linear terms were calculated in separate models [27], and linear terms were included in the models used to calculate quadratic gradients. Because the assumptions of parametric statistics may be violated in the case of survival data (live/die), which tend to have non-normally distributed errors, we computed significance values for selection gradients using logistic regression on the same models [28]. Though past studies linking morphology with habitat use have primarily focused on the importance of hind limb length alone (e.g., [8]), the action of natural selection is inherently multivariate [27,29], and studies should attempt to measure selection acting on biologically relevant trait combinations [7,30]. Our previous work on anoles has shown a strong influence of correlational selection on traits such as immune function, population density, limb length, and perch diameter [7,31] and work on other lizards demonstrates similar forms of selection on life history traits [32]. Based on previous studies [5,15], and on the ecology of *Anolis *ecomorphs [4], we predicted that hind and forelimb lengths should be selected in concert. We therefore report here the magnitude of correlational selection gradients (γ_1,2_) on size-corrected limb lengths. We regressed limb lengths on SVL (both log transformed) and used the residuals about the regression line as an estimate of size corrected limb lengths in all models. All trait distributions were standardized to have a mean of zero and unit variance, except survival, which was standardized by the population mean prior to analysis (Tables 1 and 2). We computed fitness surfaces using the non-parametric cubic spline approach of Schluter and Nychka [33]. We first searched a range of possible smoothing parameters (λ) to find the value of λ that minimized the generalized cross validation (GCV) score. We then used this λ to plot the best-fit cubic spline to survival data. Finally, because each year represents selection on a new cohort of lizards, and because of significant year effects (see Results) we treat individual years as independent estimates of selection and do not use post-hoc correction methods on significance levels.

**Table 1:** Selection gradients for all traits and all years on Kidd cay.

W (2003) 33.8% survival	β/γ	SE	χ^2^	P-Value
Hindlimb	0.09	0.14	0.57	0.45
Forelimb	0.03	0.14	0.06	0.79
Hindlimb^2^	-0.34	0.22	3.19	0.07
Forelimb^2^	-0.30	0.26	1.96	0.16
Hindlimb × Forelimb	0.293	0.16	4.14	0.04

W (2003) Kidd cay				

SVL	0.03	0.17	0.39	0.53
SVL^2^	0.16	0.20	0.57	0.45

W (2004 Animals replaced) 32% survival				

Hindlimb	-0.13	0.15	0.77	0.37
Forelimb	-0.13	0.15	0.66	0.42
Hindlimb^2^	0.14	0.20	0.49	0.48
Forelimb^2^	0.24	0.26	1.07	0.30
Hindlimb × Forelimb	0.01	0.17	0.00	0.96

W (2004)				

SVL	0.05	0.15	0.11	0.74
SVL^2^	0.60	0.22	6.88	0.009

W (2005) 45% survival				

Hindlimb	0.04	0.12	0.04	0.84
Forelimb	-0.03	0.12	0.18	0.67
Hindlimb^2^	-0.006	0.16	0.00	0.96
Forelimb^2^	0.38	0.18	4.39	0.04
Hindlimb × Forelimb	-0.13	0.18	0.52	0.47
W (2005)	β/γ	SE	χ^2^	P-Value
SVL	-0.23	0.11	4.29	0.04
SVL^2^	-0.12	0.16	0.63	0.43

W (2006) 42% survival				

Hindlimb	-0.03	0.10	0.00	0.98
Forelimb	0.13	0.10	0.44	0.50
Hindlimb^2^	0.12	0.12	0.80	0.37
Forelimb^2^	-0.10	0.14	0.75	0.38
Hindlimb × Forelimb	-0.21	0.09	5.64	0.02

W (2006)				

SVL	0.09	0.13	0.46	0.50
SVL^2^	0.02	0.18	0.02	0.88

Data distributions were standardized (by year) to mean zero and unit variance. Hind and forelimb lengths refer to residual values (regression of log-transformed limb and snout-vent-lengths. W represents survival (0/1). Linear (β) and non-linear (γ) selection gradients come from general linear models, but significance tests were computed from logistic regressions. Quadratic gradients and associated standard errors were doubled [34].

**Table 2:** Results from an experimental manipulation of vegetation on a separate study island (Nightmare cay), illustrating selection on body size but not limb length following vegetation removal (2005) and relaxed selection on body size the following year when vegetation was allowed to grow back.

W (2005 Vegetation removal) 23% survival	β/γ	SE	χ^2^	P-Value
Hindlimb	-0.12	0.23	0.30	0.58
Forelimb	0.07	0.23	0.14	0.71
Hindlimb^2^	0.40	0.46	0.41	0.52
Forelimb^2^	0.44	0.48	0.71	0.40
Hindlimb × Forelimb	-0.57	0.42	1.81	0.18

W (2005 Vegetation removal)				

SVL	0.55	0.23	8.15	0.004
SVL^2^	0.08	0.28	2.25	0.13

W (2006 Vegetation recovery) 33% survival				

Hindlimb	-0.14	0.17	0.69	0.40
Forelimb	-0.08	0.17	0.28	0.59
Hindlimb^2^	-0.28	0.28	1.03	0.31
Forelimb^2^	-0.32	0.30	1.21	0.27
Hindlimb × Forelimb	0.10	0.23	0.12	0.73

**Term**				

SVL	0.06	0.19	0.10	0.75
SVL^2^	0.20	0.22	0.79	0.37

Abbreviations and analyses as in Table 1.

## Results

**Figure 1:**
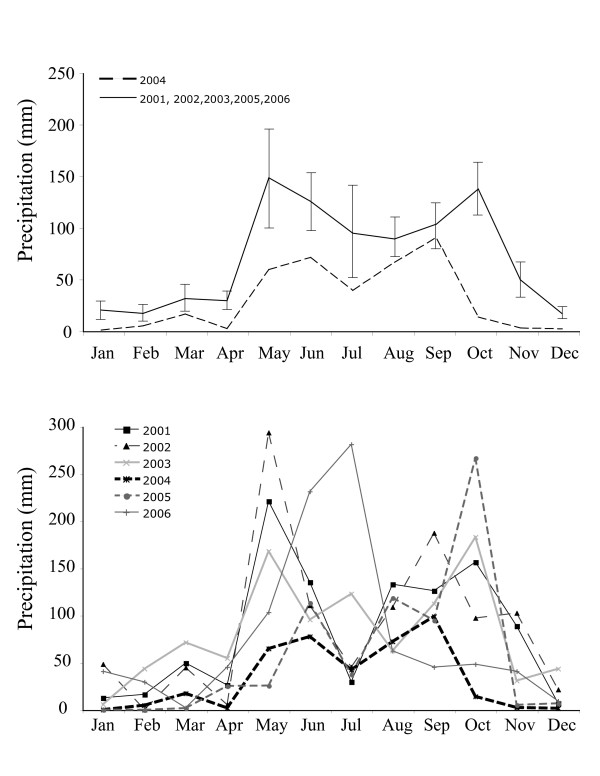
**Precipitation rates estimated from remotely-sensed measurements (TRMM) for Great Exuma, Bahamas**. The top panel depicts monthly totals [mean values (± 1 SE)] based on the wet years 2001, 2002, 2003, 2005, and 2006 (solid line) and the monthly totals for the drought year (2004; hatched line). Monthly totals for each year individually are illustrated in the bottom panel (2004 heavy dashed line).

**Figure 2:**
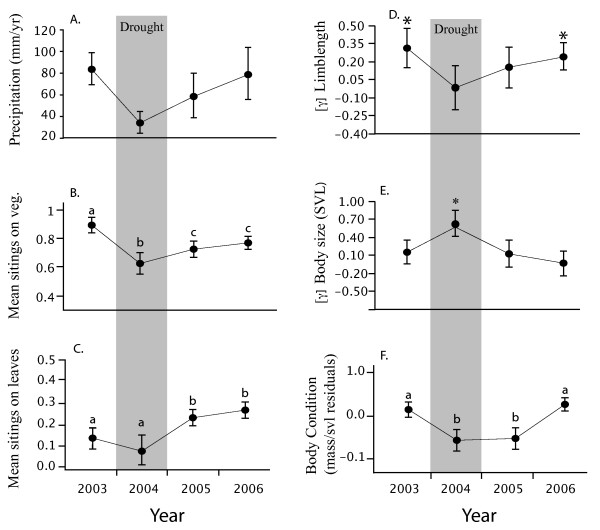
**Panel (A) shows the significantly lower rainfall totals from ground based estimates of the drought during 2004 (grey columns) which had significant impacts on habitat use by lizards (B), the frequency of leaf use for cover (C) correlational selection gradients on limb length (D) quadratic selection gradients on body size (E) and changes in mean body condition (F)**. In each panel, points represent mean values ± 1 SE. In contrast to figure 2, here we report the magnitude of selection gradients to stress the years in which selection acted on limb length or body size compared to when it did not. Significant differences in means (based on Tukey-Kramer post hoc tests) are denoted by differing lower-case letters. Asterisks denote significant selection gradients.

We began our study of natural selection during 2003. The following year we experimentally replaced male lizards on Kidd cay, our principal study site, with individuals from an adjacent site on Great Exuma [14]. Coincidentally, that same year the Bahamas experienced a severe drought that we quantified using both satellite remote sensing and ground-based rainfall data (Figs. 1, 2A). Satellite data of the entire study area corroborated the rainfall data collected at one specific point on the ground. Thus, we can be reasonably certain that our measures on the ground are representative of adjacent study sites (e.g., Nightmare cay). During 2004, under-story vegetation and canopy foliage on the island were greatly reduced, and this fine scale variation was reflected in a reduction in NDVI (see below).

Changes in vegetative cover over the course of the four-year study impacted habitat use by lizards. Lizards perched more frequently on the ground during the drought relative to wet years when they perched more frequently in the vegetation (χ^2 ^= 14.96, df = 3, P < 0.0001; Fig. 2B, C). Drought conditions ended in 2005 with an increase in rainfall (Fig. 1, 2A), which led to increased growth of under-story vegetation and shifts in habitat use by lizards (Fig. 2B, C). Use of vegetated habitat increased significantly from 2004 to 2006 though it remained below pre-drought values. Similarly, the frequency of leaf-use for perching and for cover increased during 2005 and 2006 as lizards moved off of narrow plant stems and onto new vegetative growth (χ^2 ^= 13.37, df = 3, P = 0.0003; Fig 2C).

**Figure 3:**
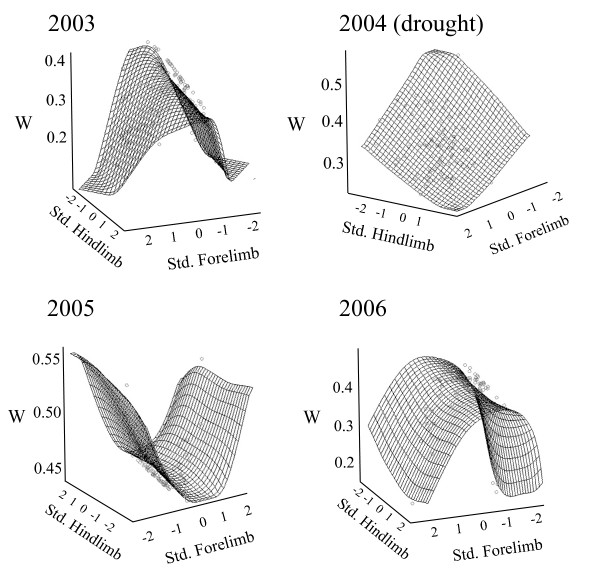
**Inter-annual variation in correlational selection acting jointly on fore and hindlimb lengths**. Surfaces were produced using the cubic spline approach developed by Shluter and Nychka [33]. The vertical axis shows the probability of survival (W) and each horizontal axis represents a standardized residual limb length. Surfaces range from convex to concave in wet years, but there was no curvature in the surface during the drought year.

Impacts of the drought on vegetation structure and habitat use by lizards coincided with shifts in natural selection on limb length and body size (snout-vent-length, SVL). During the three wet years of our study, correlational selection gradients acting on size corrected limb lengths (residuals from the regression of limb length against SVL) ranged from significant and positive to significant and negative (Fig. 3; 2003: γ_1,2_= 0.29 ± 0.16; 2005: γ_1,2_= -0.13 ± 0.18; 2006: γ_1,2 _= -0.21 ± 0.09). This indicates a change in the shape of the fitness surface from saddle shaped to dome shaped [34,35]. An alternative to focusing on the shape of the correlational selection surface (i.e., the sign of the gradients) is to ask simply whether or not selection acted on limb lengths in wet versus dry years. A plot of the absolute values of correlational selection gradients through time (Fig. 2D) shows that correlational selection gradients were significantly different from zero only during wet years. By contrast, selection operated on body size and not on limb lengths during the drought year and immediately following the drought (Fig. 2E and Table 1).

**Figure 4:**
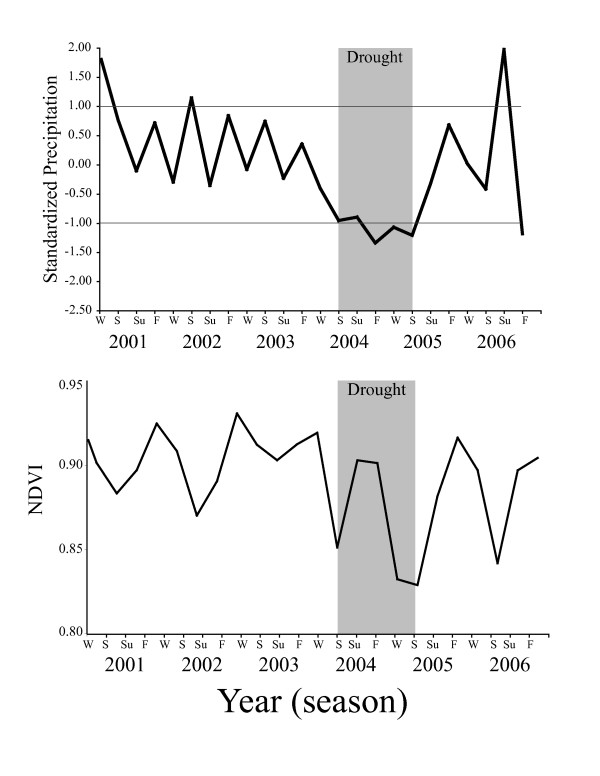
**The top panel shows seasonal variation in the TRMM (precipitation) data for each season from 2001–2006 illustrating the drought that occurred during 2004 (grey)**. Precipitation data were standardized by subtracting mean values and dividing by the standard deviation of all seasonal values. Horizontal lines at ± 1 SD highlight the magnitude of the drought. The lower panel shows corresponding changes in the untransformed "normalized difference vegetation index" (NDVI). Seasonal abbreviations reference three-month averages corresponding to winter (Dec, Jan, Feb), spring (Mar, Apr, May), summer (Jun, Jul, Aug) and fall (Sep, Oct, Nov). The grey bar highlights the drought year and is congruent with the figures from the main text.

During the drought selection on body size was significant and disruptive (γ = 0.60 ± 0.22). Following the drought, natural selection favoured smaller male body sizes (β = -0.23 ± 0.11). In the two years following the drought (Fig. 2A), the island's vegetation gradually recovered (e.g., Fig. 4 increases in NDVI,) and we observed a return to selection on limb length as lizards moved back onto the vegetation (Fig. 2E). The differences in selection on body size and limb traits was significant among years (hindlimb × forelimb × SVL × wet/dry F_1,464 _= 4.69, P = 0.03). In sum, we observed selection on limb length only in wet years, selection on body size alone during the drought, and selection for smaller body size the year immediately after the drought. Thus variation in selection was intimately related to variation in ecology.

**Figure 5:**
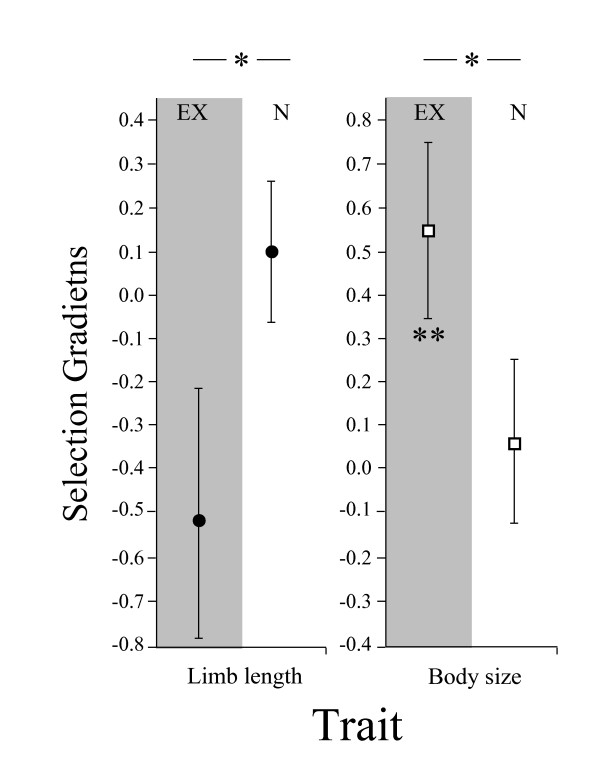
**An experimental manipulation to simulate drought effects (grey columns) on vegetation structure revealed changes in selection acting on body size that were similar to those between naturally wet and dry years (EX and N refer to experimental vegetation removal and natural years respectively)**. Following experimental removal of vegetation, natural selection became strong and directional, favoring larger male body size (** indicates the significant selection gradient). We detected no significant selection on limb length. However, comparison of slopes revealed significant differences between vegetation removed and natural years (indicated by *).

To test whether changes in habitat structure were sufficient to explain the dynamic patterns of selection observed in our natural study population, we measured selection on a separate island that we experimentally manipulated during the wet year of 2005. Similar to patterns observed during the actual drought, selection acted significantly on body size (β = 0.55 ± 0.23; Fig. 5) but not on limb morphology (γ_1,2_= -0.57 ± 0.42) following our experimental vegetation removal.

In 2006, observations in the field suggested re-growth of the under-story and substantial increases in new stem growth in the canopy on the island that we had manipulated the previous year. We again estimated selection on a new cohort of experimentally introduced males (92 juvenile male lizards from the same source-population as used the previous year, all other males were removed from the island) and similar to results from the natural site, in the year after our experimental manipulation of the vegetation, selection on body size was relaxed and non-significant (β = 0.06 ± 0.19; Fig. 5; Table 2). We did not detect significant correlational selection on limb lengths in the population where we experimentally manipulated vegetation (γ_1,2_= 0.10 ± 0.23), but for both body size and for limb length, a comparison of slopes revealed significant differences between natural populations and those in which we manipulated vegetation (body size P < 0.001 and limb length P < 0.002).

## Discussion and conclusion

Together, these results provide strong evidence that ecology can have dramatic impacts on the selective landscape through time [36]. We have demonstrated that natural selection operated on different traits in wet and dry years and that this change was associated with changes in habitat use. During wet years, when abundant vegetative habitat was available to lizards for perching, selection pressures were variable among years but operated primarily on limb traits. By contrast, during the drought, when vegetation died off and less habitat was available for perching, natural selection operated primarily on body size. This difference is likely explained by the fact that limb length is linked to variation in locomotor performance on broad versus narrow perches [7,8], whereas body size may be more important in mediating competitive interactions [37] and surviving periods of physiological stress [38,39].

Though demonstrations of natural selection in the wild have become more common in recent years, studies that convincingly illustrate the ecological basis for alternative adaptive optima are rare [3,40,41] and experimental studies such as this one, all the more so. *Anolis *lizards provide an ideal opportunity to experimentally bridge ecological changes with changes in the adaptive landscape, because the correlations between morphology, performance, and habitat use have been so thoroughly documented in this group [15]. Long hind and forelimb lengths increase sprinting speed on broad perching surfaces, but may compromise agility on narrow surfaces, or in complex habitats [6,8]. Body size is likely important in competitive interactions [9,42,43], and this may be especially true when reduced habitat availability places a premium on territoriality [9]. Large body size may also be important for surviving periods of physiological stress brought on by lack of food or water.

A limitation of our study, reflecting one of the many constraints of working in natural systems, is that the drought during 2004 coincided with our experimental replacement of male *A. sagrei *at our main study site [14]. It is therefore impossible to conclude with certainty that the observed changes in selection during 2004 arose solely due to drought, rather than our manipulation of the male lizard population. Only a second or third estimate of selection in un-manipulated populations would have allowed us to examine this. However, several lines of evidence suggest that our results are due to natural processes rather than our manipulation. First, we have previously shown that the introduction of novel male *A. sagrei *did not have a direct impact on selection dynamics [14]. Second, behavioral, climatological, and habitat data all support a dramatic effect of the drought. Finally, our experimental manipulation of the habitat during a naturally wet year provides corroborative evidence that habitat use may drive selection.

In addition to having less available habitat, lizards may have spent less time in vegetation during the drought because of changes in prey availability or predation intensity [15] (e.g., a reduction in foliage may have rendered lizards more visible to avian predators). Similarly, changes in precipitation could alter rates of parasitemia as lizards in our study populations are affected by malaria and other blood borne pathogens (Bonneaud et al., in prep). Thus, an additional caveat to consider is that our manipulations of the habitat simulated some of the effects of drought on vegetation, but could not account for potential changes in diet, disease, or predation regime. Consequently, our experimental results may underestimate differences in selection on limb length between wet and dry years.

Making associations between intra- and inter-specific variation has been a central theme of studies attempting to understand the mechanisms giving rise to the adaptive radiation of *Anolis *lizards. Implicit in this approach is the assumption that processes that gave rise to patterns of species diversity can be revealed by studying processes at the population level [12]. Phylogenetic studies of *Anolis *lizards have suggested that the same ecomorph may evolve repeatedly in response to similar environmental conditions, despite having independent evolutionary histories [11]. Our results suggest an important caveat to these findings by demonstrating that natural selection may favour different traits across years depending on environmental conditions. Sustained environmental change, such as that predicted by global climate models, could potentially drive selection pressures to act on different suites of traits [44], thereby fundamentally altering adaptive landscapes [36,44]. Under these circumstances future processes at the population level might bear little resemblance to the processes that gave rise to present interspecific variation.

## Authors' contributions

RC was responsible for the research planning, field data collection, statistical analysis, and drafting the manuscript. WB gathered and analyzed remote sensing data. TBS contributed to the research planning. All authors were involved in editing the manuscript.
